# 

**Published:** 1878-02

**Authors:** 


### CONTENTS.

### COMMUNICATIONS.

Dental Education.—*By Thomas Fillebrown, D. D. S.* .......... 45

Report on Therapeutics.—*By Frank M. Odell, D. D. S.* .......... 54

Prevention of Disease. Self-Abnegation.—*By Henry S. Chase* .......... 59

Cases of Infantile Paralysis—Paraplegia by Rheumatic Metastasis, ending in Hemiplegia and Recovery, and Cerebral Paralysis.—*By J. J. Caldwell, M. D.* .......... 60

Medical and Surgical Society of Baltimore, Md.—*By Jahn J. Caldwell, M. D* .......... 65

Retaining Points.—*By Simeon H. Guilford, A. M., D. D. S.* .......... 70

Celluloid.—*By J. B. Tullis, D. D. S.* .......... 73

Filling Teeth.—*By J. B. Tullis, D. D. S.* .......... 74

A Peculiar Case of Irritation of the Nervous System, in a child whilst Teething.—*By Dr. A. Plumberg* .......... 76

### SELECTIONS.

Man's Average Height .......... 79
Something new about Oxygen .......... 79
Removal of Strong Odors from the Hands .......... 80
The Science of Temperance .......... 81
The Student's Hysteria .......... 82
Celluloid Manufacturing Company .......... 83

### EDITORIAL.

Mississippi Valley Dental Association .......... 84
Ohio Dental College Association .......... 85
Obituary .......... 85
Obituary .......... 86
Erratum .......... 87

